# Sea-Water in Ophthalmia
*Med. and Phys. Journal for April, 1826.


**Published:** 1826-07

**Authors:** 


### SEA-WATER IN OPHTHALMIA.*

In the April Number of the Medical and Physical Journal, is a paper by Mr. Richmond, on the efficacy of sea-water in arresting the progress of ophthalmia. We shall endeavour to give a short account of it.

In 1815, an ophthalmy broke out in the 2nd battalion of the 11th regiment, then quartered at Gibraltar, which spread with great rapidity and violence. We shall not enter into the minute and unnecessary details of symptoms, as they were those that usually characterise the disease, in its acute form. There were, however, besides intense shooting pain in the eye-ball and fore-head, considerable fever and a strong hard pulse. It were needless to particularize the various *guesses* as to the cause of the epidemic, since they were all tolerably far from the mark. The principal agents in producing the disease appear to have been the strong reflected light from the batteries plaistered as they were with lime, and the limestone floors; and, added to this, the great quantities of sand and dust which were raised by the sirocco winds which blew with great violence—the atmospherical changes too must have had their share in aggravating, if not causing the malady in question. The men were likewise often employed in blasting rocks and other government works in which the eyes were much exposed to the action of particles of fire, &c.

So much for the etiology; the treatment is simple and as it seems

---

* Med. and Phys. Journal for April, 1826.

successful. It was this: that the men should bathe regularly in the sea, and that each should bring home with him some sea-water with which the eyes were to be bathed seven or eight times a day: strict cleanliness was also enjoined. These means were successful—the health and activity of the men being restored effectually, and the disease, so says the report, finally checked in eight days. But the principal treatment consisted of strict antiphlogistic regimen, venesection, frequently to syncope—strong cathartics, and constant nausea (not carried to vomiting) by small doses of tartar-emetic, aided by the warm bath. The collyria were solutions of the sulphates of zinc and alum, the latter preferable, injected every hour between the eye-lids, which prevents the purulent matter from accumulating and irritating the organ. By these remedies the disease was almost always cured in two weeks, and the sight left unimpaired.

Our author had afterwards an opportunity of putting his methodus medendi to the test, at Bombay, where ophthalmia prevailed to a great extent in the year 1823. The patients were the children of the charity-schools, and the sea-water ablutions, &c. aided by belladonna when the pupil was much contracted, were found effectual in checking the complaint. The *granulations* on the conjunctivæ were removed by the application of the sulphate of copper.

